# Associations Between Vitamin D Intake and Progression to Incident Advanced Age-Related Macular Degeneration

**DOI:** 10.1167/iovs.17-21673

**Published:** 2017-09

**Authors:** Bénédicte M. J. Merle, Rachel E. Silver, Bernard Rosner, Johanna M. Seddon

**Affiliations:** 1 University of Bordeaux, Inserm, Bordeaux Population Health Research Center, Lifelong Exposures, Health and Aging, Bordeaux, France; 2 Ophthalmic Epidemiology and Genetics Service, New England Eye Center, Tufts Medical Center, Boston, Massachusetts, United States; 3 Channing Division of Network Medicine, Harvard Medical School, Harvard University, Boston, Massachusetts, United States; 4 Department of Ophthalmology, Tufts University School of Medicine, Boston, Massachusetts, United States; 5 Sackler School of Graduate Biomedical Sciences, and Friedman School of Nutrition Science and Policy, Tufts University, Boston, Massachusetts, United States

**Keywords:** vitamin D, advanced age-related macular degeneration, epidemiology, nutrition, progression

## Abstract

**Purpose:**

There is growing evidence of the importance of nutrition in age-related macular degeneration (AMD), but no prospective studies have explored the impact of vitamin D. We evaluated the association between vitamin D intake and progression to advanced AMD.

**Methods:**

Among 2146 participants (3965 eyes), 541 (777 eyes) progressed from early or intermediate AMD to advanced disease (mean follow-up: 9.4 years) based on ocular imaging. Nutrients were log transformed and calorie adjusted. Survival analysis was used to assess associations between incident advanced disease and vitamin D intake. Neovascular disease (NV) and geographic atrophy (GA) were evaluated separately. Combined effects of dietary vitamin D and calcium were assessed based on high or low consumption of each nutrient.

**Results:**

There was a lower risk of progression to advanced AMD in the highest versus lowest quintile of dietary vitamin D intake after adjustment for demographic, behavioral, ocular, and nutritional factors (hazard ratio [HR]: 0.60; 95% confidence interval [CI]: 0.43–0.83; *P* trend = 0.0007). Similar results were observed for NV (HR: 0.59; 95% CI: 0.39–0.89; *P* trend = 0.005) but not GA (HR: 0.83; 95% CI: 0.53–1.30; *P* trend = 0.35). A protective effect was observed for advanced AMD among participants with high vitamin D and low calcium compared to the group with low levels for each nutrient (HR: 0.67; 95% CI: 0.50–0.88; *P* = 0.005). When supplement use was considered, the effect was in the protective direction but was not significant.

**Conclusions:**

A diet rich in vitamin D may prevent or delay progression to advanced AMD, especially NV. Additional exploration is needed to elucidate the potential protective role of vitamin D and its contribution to reducing visual loss.

Age-related macular degeneration (AMD) is the primary cause of irreversible vision loss in developed countries.^1,2^ The two advanced subtypes, neovascular disease (NV) and geographic atrophy (GA), are preceded by early and intermediate stages that usually have minimal visual impact but can progress to visual impairment and blindness. The prevalence of AMD is projected to increase 40% by 2040^3^ without the development of preventive and therapeutic strategies. Although effective treatments are available for NV^4^ and treatments for GA are under investigation, the overall prevention of advanced AMD remains more challenging.

The etiology of AMD is multifactorial, with genetic and modifiable factors contributing to personal risk. These modifiable factors are a target for reducing vision loss, and epidemiologic studies show that nutrients and dietary patterns influence the onset and progression of AMD. High consumption of fish,^5–9^ antioxidants,^10,11^ lutein and zeaxanthin,^10,12–16^ folate,^17^ and eicosapentaenoic acid (EPA) and docosahexaenoic acid (DHA)^5–9,18–23^ has been associated with reduced AMD risk. High adherence to a Mediterranean-style diet was also recently associated with a 26% lower risk of progression to advanced AMD.^24^ A complete understanding of dietary factors could facilitate the identification of high-risk behaviors, and assist in the implementation of preventive strategies early in the disease process.

The anti-inflammatory properties of vitamin D^25^ are of particular interest as inflammation may play a pivotal role in AMD pathology.^26,27^ A protective association between higher serum vitamin D levels and a lower prevalence of AMD was recently reported in a meta-analysis,^28^ and some studies have reported associations between dietary vitamin D and AMD.^19,29,30^ Vitamin D also shares common food sources with calcium, and calcium dysregulation has been implicated in the pathogenesis of neurodegenerative eye diseases.^31,32^ At present, however, associations between dietary calcium and AMD are inconsistent.^33,34^ In addition, the prospective relationship between dietary intake of these nutrients and advanced AMD subtypes has not been assessed in a large cohort of men and women. We therefore evaluated the association between vitamin D intake and progression to advanced disease in a large prospective AMD cohort, and hypothesized that high consumption of dietary vitamin D could help reduce incident advanced AMD.

## Methods

### Study Population and Definition of Progression

All participants were previously enrolled in our longitudinal, nationwide genetic and epidemiologic studies of AMD, which began in 1988. Participants were derived from clinic populations and nationwide referrals and were prospectively followed. This research adhered to the tenets of the Declaration of Helsinki and was performed under approved institutional review board protocol. Written informed consent was obtained for all participants.

The selection criteria for this study are displayed in Supplementary Figure S1. Participants with advanced disease in both eyes at baseline were not eligible for inclusion (*n* = 669), and those with less than 1 year of follow-up were excluded (*n* = 552). Among the 552 participants, 61 actively enrolled participants had less than 1 year of follow-up to date, 62 had no follow-up data, 95 had withdrawn from the study, and 334 were deceased prior to their initial follow-up visit. Among 2224 eligible participants, 78 were excluded for total energy intake (TEI) outside the appropriate range (defined as 600–4200 kcal for men and 600–3200 kcal for women). The final sample comprised 2146 participants (3965 eyes). The average interval between follow-up visits was 1.8 years.

Eyes were classified by ocular examination, fundus photography, and optical coherence tomography (OCT) imaging using the Clinical Age-Related Maculopathy Staging (CARMS) system.^35^ CARMS grades were defined as follows: grade 1 (no AMD, no drusen or only a few small drusen < 63 μm); grade 2 (early AMD, intermediate size drusen 63–124 μm); grade 3 (intermediate AMD, large drusen ≥ 125 μm); grade 4 (advanced dry AMD, or GA, including both central and noncentral forms); and grade 5 (advanced exudative AMD, or NV, with choroidal neovascularization). Progressors were defined as subjects who transitioned from no AMD, early AMD, or intermediate AMD to either GA or NV in at least one eye. Nonprogressors did not develop either advanced subtype during the course of the study. Progressors and nonprogressors were determined based on review of longitudinal clinical records and ocular imaging.

### Ocular Examinations and Clinical Records

An ocular examination was conducted upon enrollment. Detailed protocols and standardized clinical data forms were designed by JMS. Refraction, best-corrected visual acuity, and cataract status were assessed, intraocular pressure was measured, and iris color was classified. Color fundus photographs were obtained in up to seven standard fields based on the modified Airlie House classification, adopted by the Early Treatment Diabetic Retinopathy Study and previously described elsewhere.^36^ OCT, autofluorescence, and infrared imaging were also obtained for many participants. Baseline and prospective follow-up examination data and imaging were evaluated. JMS determined the baseline AMD grade, as well as all follow-up grades, without knowledge of dietary status or any other exposures.

### Dietary Data

Dietary data were derived from food frequency questionnaires (FFQs). The FFQs were modified from a previously existing and extensively validated questionnaire,^37^ and contained foods considered as major sources of a variety of nutrients. This questionnaire was previously demonstrated to be reliable in an age-related eye disease study population of elderly participants.^38^ All dietary data were collected upon enrollment. Participants reported the average frequency of consumption of each item during the past year. Each food was reported using standardized portion sizes, with nine possible responses, ranging from “almost never or less than once per month” to “6+ per day.” Nutrient intake was calculated at the Channing Laboratory (Harvard University School of Public Health, Boston, MA, USA), and nutrient values were derived from the United States Department of Agriculture.

Vitamin D consumption was defined as (1) dietary vitamin D intake from food sources and (2) total vitamin D intake, which is the sum of dietary and supplemental sources. Vitamin D intake was expressed in IU consumed per day. Calcium consumption was also defined according to dietary or total intake, with measurements expressed in milligrams consumed per day. The use of vitamin D supplementation was defined as “no” for subjects who reported not taking a vitamin D supplement at the time of the FFQ, and was defined as “yes” for those who reported taking a vitamin D supplement on a regular basis (at least once per week) at the time of the FFQ. The use of calcium supplementation was defined as “never” for subjects who reported never taking a calcium supplement, and was defined as “ever” for those who took a calcium supplement either currently or in the past.

### Baseline Risk Factor Data and Measurements

Demographic and behavioral data were collected using standardized questionnaires. Height, weight, and blood pressure were measured and body mass index (BMI, kg/m^2^) was calculated using height and weight measurements. The following covariates were evaluated as risk factors for progression: age (≤65, 65.1–73.9, ≥74 years), sex, education (≤high school, >high school), smoking status (never, past, current), and BMI (<25, 25–29.9, ≥30). Baseline AMD grade was determined for each eye. Nutritional intake was assessed in g/d for saturated fatty acids (SFAs), monounsaturated fatty acids (MUFAs), polyunsaturated fatty acids (PUFAs), linoleic acid (LA), EPA+DHA, lutein, and folate (μg/d).

### Statistical Analysis

Vitamin D and calcium were not normally distributed based on results from the Kolmogorov-Smirnov test for normality (all *P* < 0.01). Vitamin D (IU/d) and calcium (mg/d) were therefore log-transformed and adjusted for TEI (kcal/d) separately for men and women. These variables were normally distributed following this transformation. Calorie-adjusted vitamin D and calcium intake were ranked into sex-specific quintiles. Quintiles of nutrients were evaluated in all statistical models, with the first quintile used as the reference group.

Baseline demographic, behavioral, and ocular characteristics for progressors and nonprogressors were compared using Cox proportional hazards models. Hazard ratios (HRs) and 95% confidence intervals (CIs) for progression to advanced AMD were estimated using the eye as the unit of analysis.^39^ These comparisons were adjusted for age and sex. Associations between demographic, behavioral, ocular, and nutritional factors and quintiles of dietary and total vitamin D and calcium intake were assessed using ordinal logistic regression, adjusted for age, sex, and TEI.

The associations of dietary and total vitamin D intake with progression to advanced AMD were also analyzed using Cox proportional hazards models with the individual eye as the unit of analysis. Model 1 was adjusted for age, sex, and TEI. The full multivariate model for dietary vitamin D (model 2) was adjusted for age, sex, education, smoking status, BMI, baseline AMD grade in the study eye and the fellow eye, TEI, supplemental vitamin D use, total calcium intake, and dietary intake of folate, lutein, EPA+DHA, and MUFAs. The full model for total vitamin D intake, which includes supplement use (model 3), did not include supplemental vitamin D use as a covariate. The *P* trend was calculated using the median intake for each quintile.

Dietary and total calcium intakes were assessed using this methodology with similar adjustments to those described above. The full multivariate model for dietary calcium intake was adjusted for supplemental calcium use and total vitamin D intake (model 2). The full model for total calcium intake, which includes supplement use (model 3), did not include supplemental calcium use as a covariate. All analyses were performed using SAS 9.4 (SAS Institute, Cary, NC, USA), and *P* < 0.05 was considered statistically significant.

### Secondary Analyses

Multivariate associations between vitamin D intake, calcium intake, and progression to GA and NV were evaluated using survival analysis as described above. The combined effect of vitamin D and calcium was assessed, with high and low intake defined as greater than or equal to the sex-specific median or below the sex-specific median, respectively. Subjects were classified into four categories (for vitamin D and calcium, respectively): (1) low/low; (2) low/high; (3) high/low; and (4) high/high. The low/low group was used as the referent (see Table 5). The association between combined vitamin D and calcium intake and progression to advanced AMD was evaluated using Cox proportional hazards models as described above. Model 1 was adjusted for age, sex, education, smoking, BMI, AMD grade at baseline, and TEI. The full model for dietary intake (model 2) included all covariates listed above, as well as dietary intake of folate, lutein, EPA+DHA, MUFAs, and supplemental vitamin D and calcium use. The full model for total intake (model 3) did not include supplemental vitamin D or calcium use as covariates. The interaction between vitamin D and calcium intake was assessed. Each interaction term was introduced into the fully adjusted Cox proportional hazards model.

## Results

Among 2146 subjects, 541 (25.2%) progressed to advanced disease and 1605 did not progress (777 and 3188 eyes, respectively). The mean follow-up time was 9.4 years (range, 1.0–24.9 years). The interquartile range for this interval was 4.6 to 13.6 years, representing the follow-up time for 50% of our cohort. A Kaplan-Meier curve depicting the cumulative incidence of advanced AMD over time is presented in the Figure. The cumulative incidence of advanced disease was 10.3% (*n* = 214) at 4 years and 23.1% (*n* = 411) at 10 years.

**Figure:**
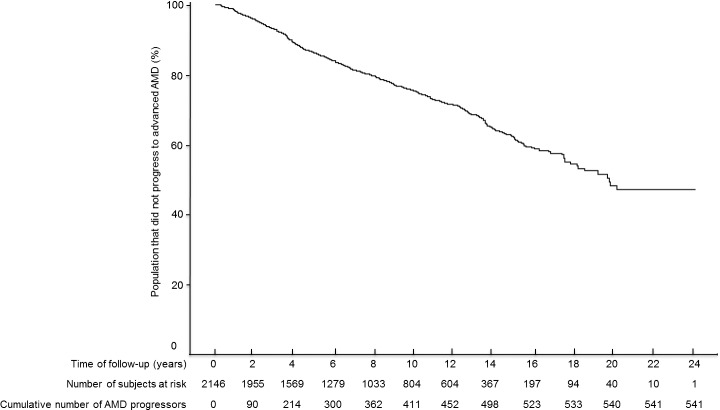
Kaplan-Meier curve for the probability of not developing advanced AMD by follow-up time.

### Associations Between Demographic, Behavioral, and Ocular Covariates and Progression to Advanced AMD

Progression to advanced AMD was significantly associated with older age (*P* trend < 0.0001), female sex (*P* trend < 0.0001), smoking (*P* trend = 0.0007), and more advanced grade at baseline (*P* trend < 0.0001). A higher level of education was protective against progression (*P* trend = 0.006) (Table 1). Although the effect estimates trended in the expected direction of an adverse effect of higher BMI, BMI was not significantly associated with progression to advanced AMD (*P* trend = 0.33).

**Table 1:**
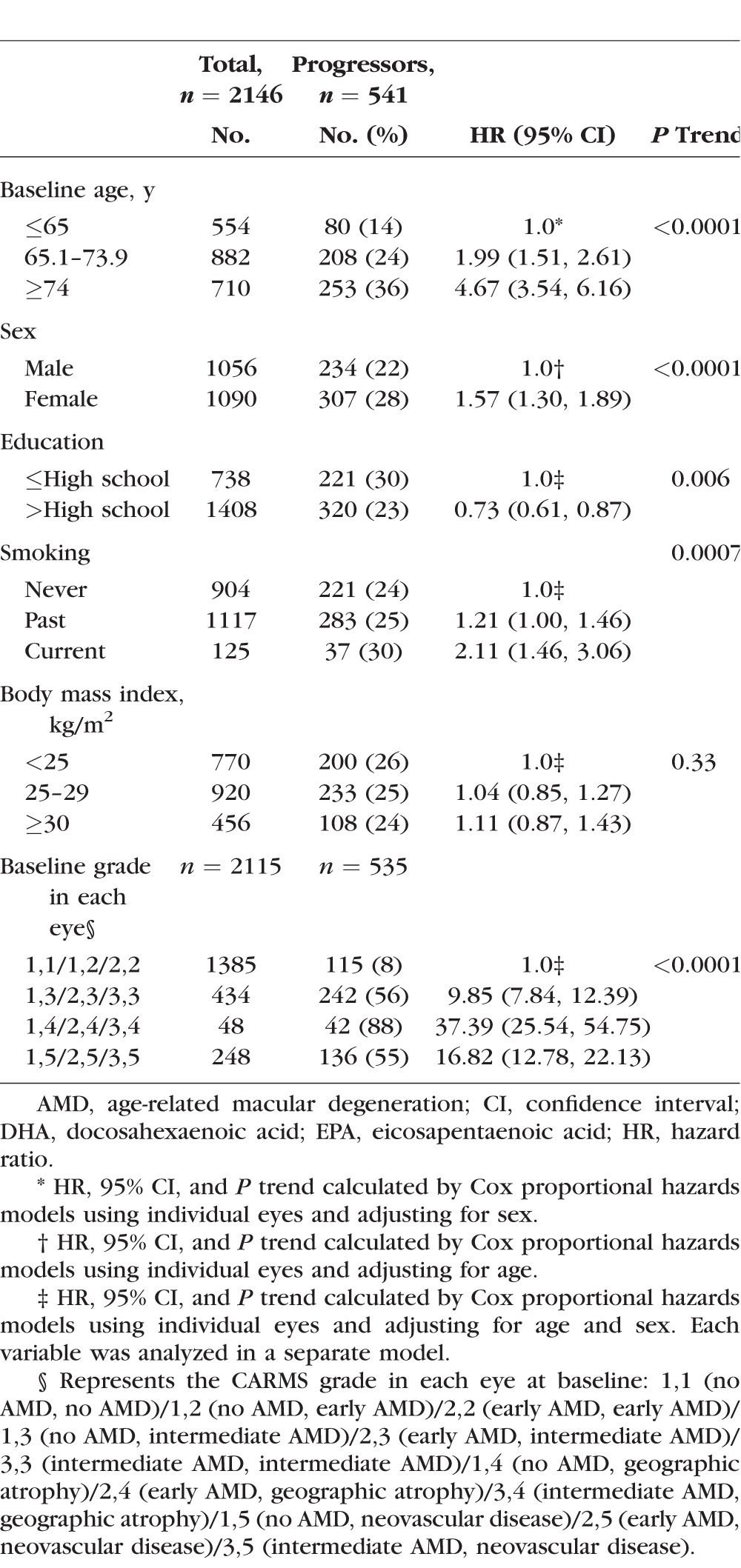
Baseline Demographic, Behavioral, and Ocular Characteristics Among Progressors and Nonprogressors to Overall Advanced AMD

### Vitamin D Intake and AMD

Subjects with a higher dietary vitamin D intake tended to be older (*P* trend = 0.0002), have a higher level of education (*P* trend = 0.003), have no history of smoking (*P* trend = 0.0003), have a lower intake of MUFAs, PUFAs, and LA (all *P* trend < 0.0001), and have a higher intake of EPA+DHA, folate, and lutein (all *P* trend < 0.0001) (Table 2). Sex, BMI, and baseline AMD grade were not significantly associated with dietary vitamin D consumption. Subjects with higher total vitamin D intake (dietary and supplemental) tended to have a higher level of education (*P* trend < 0.0001), a lower intake of MUFAs (*P* trend = 0.04) and LA (*P* trend = 0.02), and higher intake of EPA+DHA (*P* trend < 0.0001), folate (*P* trend = 0.0005), and lutein (*P* trend < 0.0001). Age, sex, smoking, BMI, AMD grade at baseline, and the intake of MUFAs and PUFAs were not significantly associated with total vitamin D.

**Table 2:**
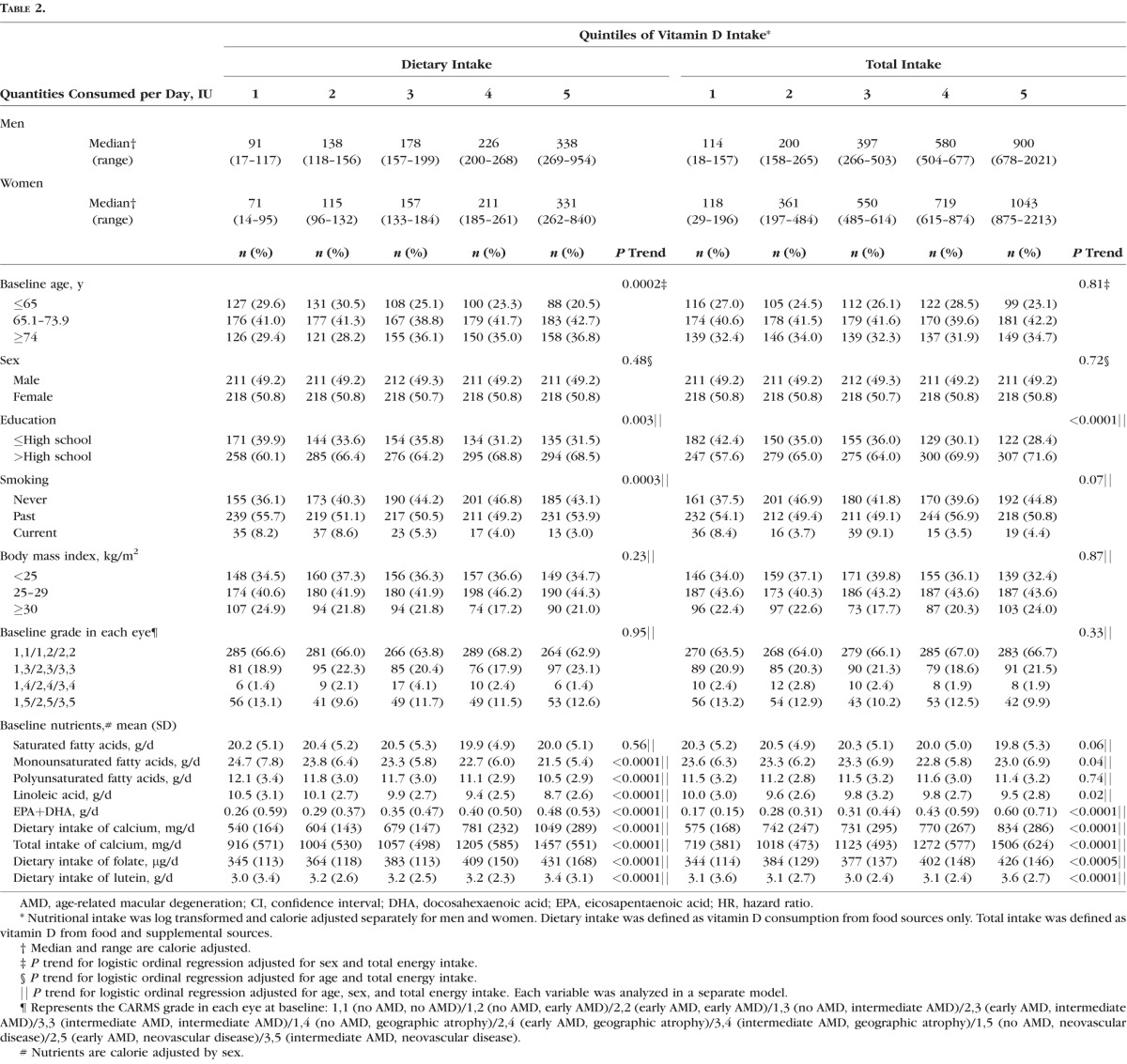
Baseline Demographic, Behavioral, and Ocular Characteristics According to Vitamin D Intake

Higher dietary vitamin D intake was significantly associated with a reduced risk of progression to advanced AMD (*P* trend = 0.0007) (Table 3), after adjustment for age, sex, education, smoking, BMI, baseline AMD grade, TEI, supplemental vitamin D use, dietary and supplemental calcium intake, and the dietary intake of EPA+DHA, MUFAs, folate, and lutein. Subjects in quintiles 4 (HR: 0.59; 95% CI: 0.43–0.80) and 5 (HR: 0.60; 95% CI: 0.43–0.83) had a significantly lower risk of progression compared to quintile 1. A similar inverse trend was observed for quintiles 2 and 3, although this result was not statistically significant. The protective effect of vitamin D observed in model 2 was primarily driven by the inclusion of total calcium intake as a covariate. We found no association between total vitamin D intake and progression to advanced AMD.

**Table 3:**
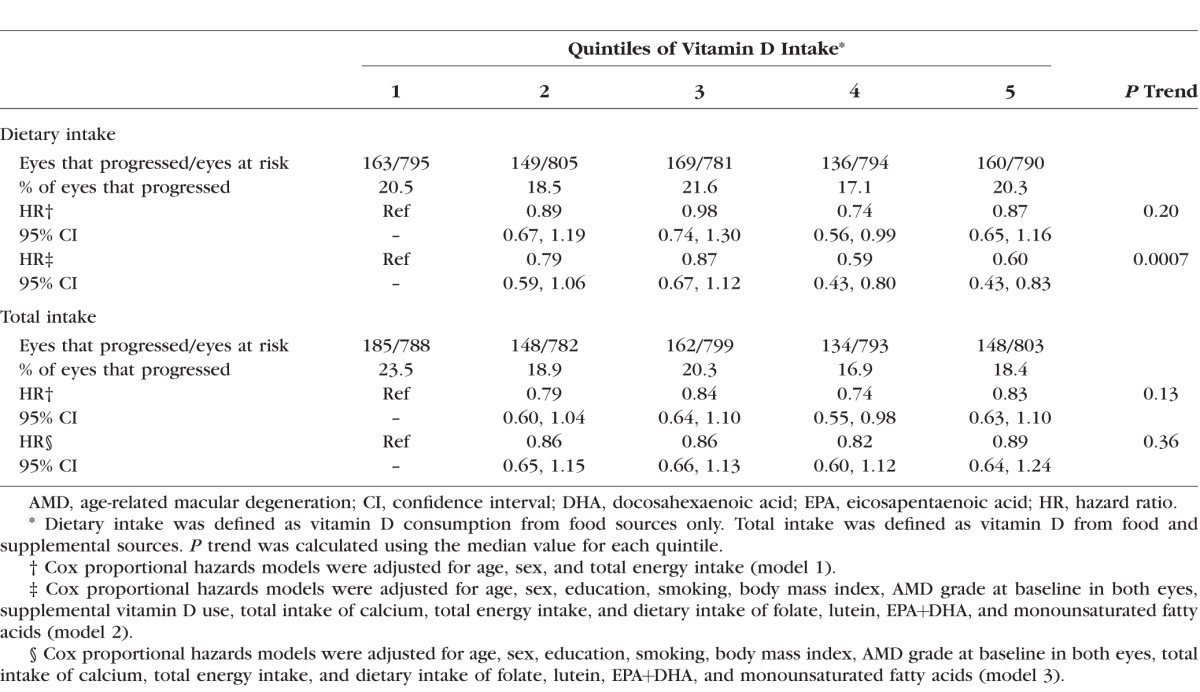
Multivariate Associations Between Vitamin D Intake and Progression to Advanced Age-Related Macular Degeneration

Higher dietary vitamin D intake was significantly associated with a reduced risk of progression to NV (HR [quintile 5 versus quintile 1]: 0.59; 95% CI: 0.39–0.89; *P* trend = 0.005), similar to results observed for overall progression. Dietary vitamin D intake was not significantly associated with progression to GA (*P* trend = 0.35). Total vitamin D intake was not associated with either advanced endpoint (Table 4).

**Table 4:**
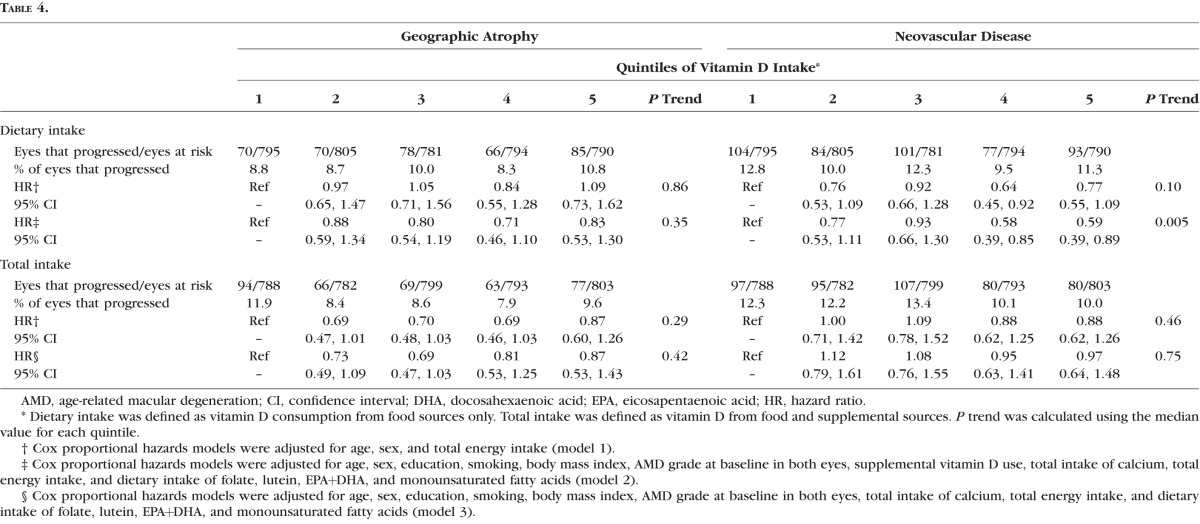
Multivariate Associations Between Vitamin D Intake and Progression to Geographic Atrophy and Neovascular Disease

### Calcium Intake and AMD

Subjects with higher calcium intake (dietary and total) tended to have a higher level education (*P* trend < 0.0001), no history of smoking (*P* trend < 0.0001), lower intake of MUFAs, PUFAs, and LA, and a higher intake of EPA+DHA, folate, and lutein (all *P* trend < 0.0001) (Supplementary Table S1). Age, sex, BMI, AMD grade at baseline, and SFA consumption were not significantly associated with calcium intake.

Subjects in quintiles 2, 3, and 4 of dietary calcium intake had a significantly lower risk of progression to AMD compared to those in quintile 1 with adjustment for age, sex, and TEI. However, after adjustment for all demographic, behavioral, ocular, and nutritional covariates, these associations were no longer significant. Subjects in the highest quintiles of total calcium intake had a higher risk of progression to advanced AMD, but the trend was not significant. Calcium intake was not significantly associated with progression to GA or to NV (Supplementary Tables S2, S3).

### Associations Between Vitamin D, Calcium, and AMD

Subjects with a high dietary vitamin D and low dietary calcium intake, representing 12.7% of our sample, had a significantly lower risk of progression to advanced AMD compared to those with low intake of both dietary vitamin D and calcium (HR: 0.67; 95% CI: 0.50–0.88; *P* trend = 0.005) after adjustment for all covariates. A suggestive beneficial effect was observed for high levels of both nutrients (HR: 0.81; 95% CI: 0.65–1.02; *P* trend = 0.07) (Table 5). There was no effect modification of vitamin D by level of calcium intake (*P* interaction = 0.63 and 0.85 for dietary intake and total intake, respectively).

**Table 5:**
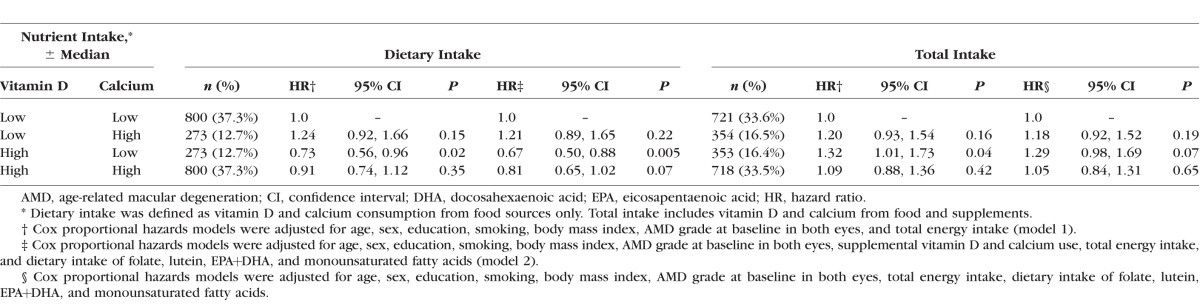
Associations Between Combined Vitamin D and Calcium Intake and Progression to Overall Advanced Age-Related Macular Degeneration

## Discussion

In this large prospective cohort study with a mean follow-up time of over 9 years, high dietary intake of vitamin D was significantly associated with a 40% lower risk of progression to advanced AMD. This association was stronger among subjects with both a high dietary intake of vitamin D and a low dietary intake of calcium. Results were independent of major known or potential AMD risk factors, including demographic, behavioral, and ocular characteristics. The beneficial effect of dietary vitamin D was observed primarily for the NV subtype. To our knowledge, this is the first prospective study to explore the impact of dietary vitamin D intake on the transition from early and intermediate stages of disease to advanced AMD, with emphasis on individual advanced disease subtypes.

We previously reported that monozygotic twins with a more advanced stage of AMD had lower vitamin D intake compared to their co-twin with earlier stages of AMD,^19^ which is consistent with our results in this longitudinal study. Also in accordance with our results, a case–control study suggested a beneficial effect of dietary vitamin D for NV,^30^ and the cross-sectional CAREDS reported a significantly lower intake of dietary vitamin D among women with early AMD compared to women with no AMD,^29^ although results were not reported for the small sample with advanced AMD (*n* = 26). A short retrospective report evaluating ICD-9 codes from Medicare claims data suggested no significant association between vitamin D deficiency and occurrence of AMD.^40^

Our study expands on previous results by exploring the prospective associations of dietary and supplemental vitamin D intake with progression to advanced AMD subtypes over 25 years in a large cohort and a high number of progressors to advanced disease. Participants in the fourth and the fifth quintiles of vitamin D intake had a similar reduced risk of progression to both overall advanced AMD and the NV subtype compared to individuals with a lower intake. This finding suggests a threshold effect, with a benefit of consuming at least 226 and 211 IU dietary vitamin D per day for men and women, respectively, and no effect of consuming smaller quantities of this nutrient. The Recommended Dietary Allowance of vitamin D is 600 IU/d for men and women up to 51 years, and 800 IU/d for age 71 and older.^41^ In our study, participants in the fourth and fifth quintiles of dietary plus supplemental vitamin D intake reported a daily consumption in accordance with these guidelines.

Vitamin D can be synthesized in the human skin upon exposure to ultraviolet light from the sun, and is otherwise obtained through the diet. Our study focused on diet, and the two major sources of vitamin D were dairy products and oily fish. In the United States, milk and dairy products are voluntarily fortified (385 IU/L) with vitamin D. Consumption of oily fish, a good source of n-3 PUFAs and vitamin D, is also known to be related to a decreased risk of AMD.^5–9,20^ The NHANES^42^ and more recently the BMES^34^ suggest that consumption of dairy products may help to reduce the risk of advanced AMD. Other foods containing high levels of vitamin D include egg yolks, lean pork, liver, and mushrooms. In our study, total vitamin D intake (dietary plus supplemental) was not significantly associated with a reduced risk of progression to advanced AMD, although the estimates of effect were in the protective direction. This result could be explained by the fact that vitamin D from food may differ from supplemental vitamin D with regard to its bioavailability, or other correlates of vitamin D beyond what we analyzed might be related to the reduction in AMD risk.

Few studies have reported associations between dietary calcium and AMD, and results are inconsistent. Similar to our results in this prospective study, the NHANES cross-sectional study reported that higher supplementary calcium consumption was associated with an increased prevalence of AMD,^33^ whereas the BMES suggested that a lower consumption of dietary calcium over 15 years was associated with a modest increase in advanced AMD risk (*n* = 84 advanced cases).^34^

The biological basis for health benefits related to vitamin D is well documented, and involves its anti-inflammatory role and the presence of vitamin D receptors in retinal tissues.^43,44^ Studies show that vitamin D reduces the production of inflammatory markers such as C-reactive protein and homocysteine,^45^ both of which have been linked to AMD risk.^26,46^ In our study, we found that high dietary intake of vitamin D was associated with a reduced risk of NV, the advanced subtype involving growth of new blood vessels in the retina, although it was not associated with GA. It is possible that vitamin D could protect against NV through its antiangiogenic properties.^47^

Strengths of our study include standardized data collection for a large cohort, classification of disease based on clinical examination and ocular imaging, extensive follow-up time, a high number of subjects who progressed to advanced AMD (allowing us to evaluate NV and GA separately), and the collection of comprehensive data related to major dietary and other behavioral confounders. The prospective design also minimized recall bias. Our analyses evaluated individual eyes rather than individual participants, accounting for eye-specific covariates and distinguishing between subjects who progress in a single eye compared with those who progress in both eyes. The use of this methodology results in an increase in statistical power. Dietary data were collected prior to the onset of advanced AMD, and the potential for dietary changes resulting from knowledge of the disease or the induced disability was therefore minimized. Our findings are applicable to an older American Caucasian population and could also be relevant for other developed countries.

Data collected from FFQs may result in an over- or underestimation of the nutrients consumed; however, all subjects are ranked using the same dietary assessment. Residual confounding is also a limitation in epidemiologic studies, and the potential benefit of vitamin D might be explained by other factors. For instance, subjects with high dietary vitamin D consumption are more likely to have a healthier lifestyle. In nutritional epidemiology, intercorrelations between nutrients cannot be completely eliminated. We therefore adjusted for numerous diet- and AMD-related risk factors in order to minimize the possibility of residual confounding from unknown factors that may have influenced dietary intake, although there were minimal changes to the results after adjustment.

Our prospective study suggests that higher intake of dietary vitamin D is associated with a lower risk of progression to advanced AMD, and especially to the NV subtype. Consuming foods rich in vitamin D may contribute to an eye-healthy diet. Additional research is needed to explore the underlying mechanisms and to better understand the potential role of vitamin D during the progression from early and intermediate disease to advanced stages of AMD.

## Supplementary Material

Supplement 1

Supplement 2
