# Supplementary figures and images for: Associations Between Vitamin D Intake and Progression to Incident Advanced Age-Related Macular Degeneration
Source: Invest Ophthalmol Vis Sci. 2017 Sep;58(11):4569–78. doi: 10.1167/iovs.17-21673 (PMC5595226; doi:10.1167/iovs.17-21673)

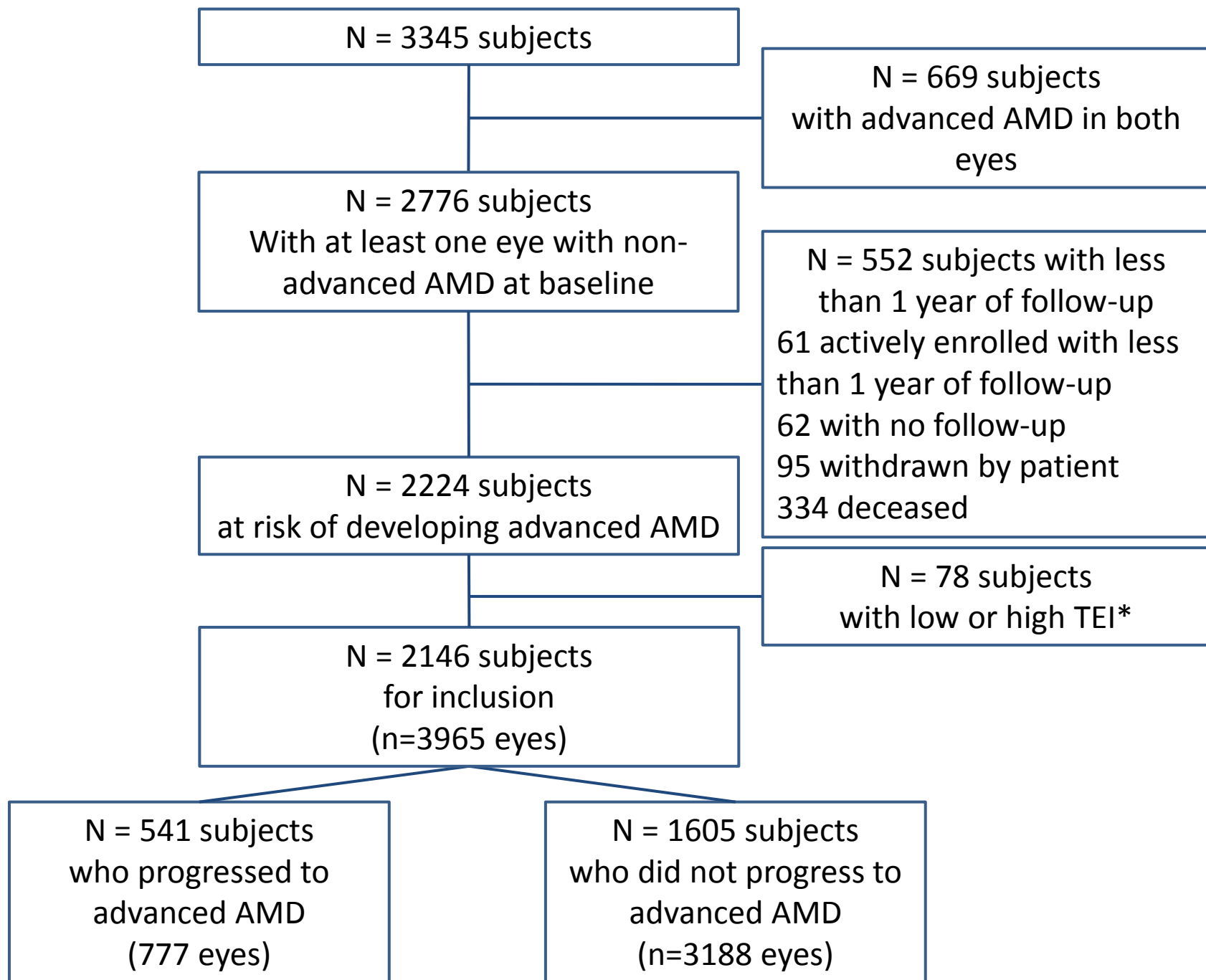

Supplement: Supplement 1 [file iovs-58-10-21_s01.pdf]
