# Supplementary material for: Associations Between Vitamin D Intake and Progression to Incident Advanced Age-Related Macular Degeneration
Source: Invest Ophthalmol Vis Sci. 2017 Sep;58(11):4569–78. doi: 10.1167/iovs.17-21673 (PMC5595226; doi:10.1167/iovs.17-21673)
Supplement: Supplement 2 [file iovs-58-10-21_s02.pdf]

Supplementary Table S1. Baseline demographic, behavioral and ocular characteristics according to dietary intake of calcium.

	Quintiles of Calcium Intake[*]											
	Dietary intake						Total intake					
**Quantities consumed per day** (mg)[+]	**1**	**2**	**3**	**4**	**5**		**1**	**2**	**3**	**4**	**5**	
Men,												
Median	448	577	682	808	1100		500	666	830	1066	1547	
(Range)	(212-523)	(524-629)	(630-733)	(734-919)	(920-3220)		(226-589)	(589-732)	(733-929)	(929-1224)	(1225-3876)	
Women												
Median	436	563	679	81	1070		571	884	1210	1614	2119	
(Range)	(249-506)	(507-622)	(623-740)	(741-918)	(919-2420)		(269-734)	(737-1045)	(1046-1387)	(1388-1803)	(1804-4439)	
	**n (%)**	**n (%)**	**n (%)**	**n (%)**	**n (%)**	***P* trend**	**n (%)**	**n (%)**	**n (%)**	**n (%)**	**n (%)**	***P* trend**
**Baseline Age** (years)						*0.82*[‡]						*0.95*[‡]
≤65	111 (25.9)	107 (24.9)	101 (23.5)	134 (31.2)	101 (23.5)		114 (26.6)	112 (26.1)	109 (25.4)	100 (23.3)	119 (27.8)	
65-74	185 (43.1)	177 (41.3)	169 (39.3)	175 (40.8)	176 (41.0)		185 (43.1)	159 (37.1)	187 (43.5)	181 (42.2)	170 (39.6)	
≥74	133 (31.0)	145 (33.8)	160 (37.2)	120 (28.0)	152 (35.5)		130 (30.0)	158 (36.8)	134 (31.2)	148 (34.5)	140 (32.6)	
**Sex**						*0.99*[§]						*0.92*[§]
Male	211 (49.2)	211 (49.2)	212 (49.3)	211 (49.2)	211 (49.2)		211 (49.2)	211 (49.2)	211 (49.2)	211 (49.2)	211 (49.2)	
Female	218 (50.8)	218 (50.8)	218 (50.7)	218 (50.8)	218 (50.8)		218 (50.8)	218 (50.8)	218 (50.8)	218 (50.8)	218 (50.8)	
**Education**						*<0.0001*[‖]						*<0.0001*[‖]
≤high school	190 (44.3)	144 (33.6)	146 (34.0)	129 (30.1)	129 (30.1)		172 (40.1)	172 (40.1)	134 (31.2)	138 (32.2)	122 (28.4)	
>high school	239 (55.7)	285 (66.4)	284 (66.0)	300 (69.9)	300 (69.3)		257 (59.9)	257 (59.9)	296 (68.8)	291 (67.8)	307 (71.6)	
**Smoking**						*<0.0001*[‖]						*<0.0001*[‖]
Never	154 (35.9)	166 (38.7)	196 (45.6)	199 (46.4)	189 (44.1)		139 (32.4)	183 (42.7)	200 (46.5)	196 (45.7)	186 (43.4)	
Past	231 (53.8)	235 (54.8)	217 (50.4)	208 (48.5)	226 (52.7)		245 (57.1)	225 (52.2)	209 (48.6)	219 (51.0)	220 (51.3)	
Current	44 (10.3)	28 (6.5)	17 (4.0)	22 (5.1)	14 (3.2)		45 (10.5)	22 (5.1)	21 (4.9)	14 (3.3)	23 (5.4)	
**Body Mass Index** (kg/m^2)						*0.38*[‖]						*0.73*[‖]
<25	159 (37.1)	164 (38.2)	158 (36.8)	138 (32.2)	151 (35.2)		144 (33.6)	159 (37.0)	151 (35.1)	160 (37.3)	156 (36.4)	
25-29	186 (43.3)	166 (38.7)	179 (41.6)	203 (47.3)	186 (43.4)		195 (45.4)	178 (41.5)	181 (42.1)	186 (43.4)	180 (42.0)	
≥30	84 (19.6)	99 (23.1)	93 (21.6)	88 (20.5)	92 (21.4)		90 (21.0)	92 (21.5)	98 (22.8)	83 (19.3)	93 (21.6)	
**Baseline Grade in Each Eye**[¶]						*0.39*[‖]						*0.29*[‖]
1,1/1,2/2,2	261 (61.4)	275 (64.6)	285 (67.7)	287 (68.2)	277 (65.6)		270 (63.8)	280 (66.0)	274 (64.9)	285 (67.9)	276 (64.8)	
1,3/2,3/3,3	104 (24.5)	83 (19.5)	75 (17.8)	84 (19.9)	88 (20.9)		88 (20.8)	90 (21.2)	84 (19.9)	70 (16.7)	102 (23.9)	
1,4/2,4/3,4	9 (2.1)	12 (2.8)	8 (1.9)	10 (2.4)	9 (2.1)		6 (1.4)	9 (2.1)	14 (3.3)	6 (1.4)	13 (3.1)	
1,5/2,5/3,5	51 (12.0)	56 (13.1)	53 (12.6)	40 (9.5)	48 (11.4)		59 (14.0)	45 (10.6)	50 (11.9)	59 (14.0)	35 (8.2)	
**Baseline**												

**nutrients[#],** mean (std)												
Saturated fatty acids, g/d	19.8 (4.7)	20.3 (5.3)	20.1 (5.3)	20.2 (5.1)	20.5 (5.2)	*0.19*[‖]	20.3 (5.1)	20.4 (5.2)	20.4 (5.1)	19.8 (4.9)	20.0 (5.3)	*0.14*[‖]
Monounsaturated fatty acids, g/d	25.0 (7.6)	24.4 (6.7)	23.1 (5.8)	22.2 (6.0)	21.2 (5.0)	*<0.0001*[‖]	24.5 (7.2)	23.3 (6.3)	23.4 (6.3)	22.4 (6.1)	22.4 (5.8)	*<0.0001*[‖]
Polyunsaturated fatty acids, g/d	12.2 (3.3)	11.9 (3.1)	11.6 (2.9)	11.1 (3.0)	10.4 (2.9)	*<0.0001*[‖]	11.9 (3.5)	11.4 (2.9)	11.6 (3.0)	11.2 (3.0)	11.1 (3.0)	*<0.0001*[‖]
Linoleic acid, g/d	10.4 (3.0)	10.2 (2.8)	9.9 (2.6)	9.4 (2.6)	8.7 (2.6)	*<0.0001*[‖]	10.3 (3.2)	9.7 (2.6)	9.9 (2.7)	9.5 (2.7)	9.2 (2.6)	*<0.0001*[‖]
EPA+DHA, g/d	0.32 (0.53)	0.33 (0.45)	0.36 (0.49)	0.40 (0.58)	0.37 (0.45)	*<0.0001*[‖]	0.27 (0.39)	0.31 (0.49)	0.33 (0.41)	0.38 (0.47)	0.49 (0.68)	*<0.0001*[‖]
Dietary intake of vitamin D, IU/d	118 (77)	145 (63)	172 (73)	208 (86)	314 (116)	*<0.0001*[‖]	133 (80)	162 (73)	186 (85)	224 (112)	251 (137)	*<0.0001*[‖]
Dietary + supplemental intake of vitamin D mg/d	391 (320)	442 (320)	491 (319)	574 (354)	654 (345)	*<0.0001*[‖]	267 (238)	411 (273)	517 (312)	611 (321)	747 (356)	*<0.0001*[‖]
Dietary intake of folate, µg/d	334 (106)	364 (107)	383 (120)	413 (146)	439 (173)	*<0.0001*[‖]	339 (99)	366 (118)	395 (139)	405 (147)	427 (160)	*<0.0001*[‖]
Dietary intake of lutein, g/d	2.5 (2.0)	3.2 (2.9)	3.1 (2.1)	3.5 (3.3)	3.5 (3.4)	*<0.0001*[‖]	2.7 (2.4)	2.9 (2.5)	3.4 (2.9)	3.3 (2.5)	3.6 (3.4)	*<0.0001*[‖]

[*] Nutritional intake was log transformed calorie-adjusted separately for men and women. Dietary intake was defined as calcium consumption from food sources only. Total intake was defined as calcium from food and supplemental sources.

[+] Median and range are calorie-adjusted.

[‡] *P* trend for logistic ordinal regression adjusted for sex and total energy intake.

[§] *P* trend for logistic ordinal regression adjusted for age and total energy intake.

[‖] *P* trend for logistic ordinal regression adjusted for age, sex, and total energy intake. Each variable was analyzed in a separate model.

[¶] Represents the CARMS grade in each eye at baseline:

1,1 (no AMD, no AMD)/1,2 (no AMD, early AMD)/2,2 (early AMD, early AMD)/1,3 (no AMD, intermediate AMD)/2,3 (early AMD, intermediate AMD)/3,3 (intermediate AMD, intermediate AMD)/1,4 (no AMD, geographic atrophy)/2,4 (early AMD, geographic atrophy)/3,4 (intermediate AMD, geographic atrophy)/1,5 (no AMD, neovascular AMD)/2,5 (early AMD, neovascular AMD)/3,5 (intermediate AMD, neovascular AMD).

[#] Nutrients are calorie-adjusted by sex.

AMD: age-related macular degeneration; CI: Confidence interval; DHA: docosahexaenoic acid; EPA eicosapentaenoic acid; HR: Hazard Ratio

Supplementary Table S2. Multivariate associations between calcium intake and progression to advanced AMD.

	Quintiles of calcium intake[*]					
	1	2	3	4	5	*P* trend
**Dietary intake**						
Eyes that progressed / eyes at risk	189/794	147/787	141/790	137/800	163/794	
Eyes that progressed (%)	23.8	18.7	17.8	17.1	20.5	
HR[+]	Ref	0.74	0.70	0.74	0.79	*0.26*
95% CI	-	0.57,0.97	0.53,0,93	0.56,0.98	0.60,1.04	
HR[‡]	Ref	0.91	1.00	0.83	1.01	*0.96*
95% CI	-	0.70,1.19	0.76,1.32	0.61,1.12	0.74,1.37	
**Total intake**						
Eyes that progressed / eyes at risk	145/787	172/799	160/788	141/784	159/807	
Eyes that progressed (%)	17.6	20.3	19.3	17.8	19.0	
HR[+]	Ref	1.07	1.14	0.87	1.10	*0.91*
95% CI	-	0.80,1.42	0.86,1.52	0.64,1.17	0.82,1.47	
HR[§]	Ref	1.28	1.35	1.11	1.40	*0.35*
95% CI	-	0.95,1.72	0.99,1.83	0.80,1.52	1.02,1.92	

[*] Nutritional intake was log transformed calorie-adjusted separately for men and women. Dietary intake was defined as calcium consumption from food sources only. Total intake was defined as calcium from food and supplemental sources. *P* trend was calculated using the median value for each quintile.

[+] Cox proportional hazards models were adjusted for age, sex, and TEI (Model 1).

[‡]Cox proportional hazards models were adjusted for age, sex, education, smoking, BMI, AMD grade at baseline in both eyes, supplemental calcium use, total vitamin D intake, total energy intake, and dietary intake of folate, lutein, EPA+DHA, and monounsaturated fatty acids (Model 2).

[§] Cox proportional hazards models were adjusted for age, sex, education, smoking, BMI, AMD grade at baseline in both eyes, total intake of vitamin D, total energy intake, and dietary intake of folate, lutein, EPA+DHA, and monounsaturated fatty acids (Model 3).

AMD: age-related macular degeneration; CI: Confidence interval; DHA: docosahexaenoic acid; EPA eicosapentaenoic acid; HR: Hazard Ratio

Supplementary Table S3. Multivariate associations between dietary intake of calcium and progression to geographic atrophy and neovascular disease.

	Geographic Atrophy						Neovascular Disease					
	Quintiles of calcium intake*											
	1	2	3	4	5	*P* trend	1	2	3	4	5	*P* trend
**Dietary intake**												
Eyes that progressed / eyes at risk	95/794	68/787	55/790	67/800	84/784		103/794	87/787	93/790	81/800	95/794	
Eyes that progressed (%)	12.0	8.6	7.0	8.4	10.6		13.0	11.1	11.8	10.1	12.0	
HR⁺	Ref	0.68	0.55	0.73	0.82	*0.65*	Ref	0.83	0.88	0.80	0.84	*0.44*
95% CI	-	0.47,0.99	0.37,0.83	0.49,1.08	0.56,1.20		-	0.60,1.17	0.63,1.24	0.57,1.14	0.60,1.18	
HR‡	Ref	0.80	0.69	0.77	1.00	*0.87*	Ref	1.07	1.28	0.98	1.05	*0.96*
95% CI	-	0.54,1.17	0.45,1.05	0.50,1.18	0.65,1.56		-	0.78,1.49	0.90,1.82	0.69,1.41	0.72,1.53	
**Total intake**												
Eyes that progressed / eyes at risk	74/787	81/799	72/788	64/784	78/807		79/787	103/799	101/788	87/784	89/807	
Eyes that progressed (%)	9.4	10.1	9.1	8.2	9.7		9.5	12.2	12.4	10.9	10.7	
HR⁺	Ref	0.99	1.01	0.79	1.05	*0.79*	Ref	1.17	1.31	1.00	1.11	*0.97*
95% CI	-	0.67,1.47	0.68,1.50	0.52,1.20	0.71,1.56		-	0.82,1.67	0.92,1.86	0.69,1.45	0.77,1.60	
HR§	Ref	1.09	1.04	0.95	1.17	*0.84*	Ref	1.37	1.62	1.37	1.40	*0.33*
95% CI	-	0.73,1.63	0.68,1.60	0.61,1.48	0.74,1.84		-	0.95,1.96	1.12,2.33	0.91,2.04	0.92,2.12	

* Nutritional intake was log transformed calorie-adjusted separately for men and women. Dietary intake was defined as calcium consumption from food sources only. Total intake was defined as calcium from food and supplemental sources. *P* trend was calculated using the median value for each quintile.

⁺Cox proportional hazards models were adjusted for age, sex, and TEI (Model 1).

‡ Cox proportional hazards models were adjusted for age, sex, education, smoking, BMI, AMD grade at baseline in both eyes, supplemental calcium use, total intake of vitamin D, total energy intake, and dietary intake of folate, lutein, EPA+DHA, and monounsaturated fatty acids (Model 2).

§ Cox proportional hazards models were adjusted for age, sex, education, smoking, BMI, AMD grade at baseline in both eyes, total intake of vitamin D, total energy intake, and dietary intake of folate, lutein, EPA+DHA, and monounsaturated fatty acids (Model 3).

AMD: age-related macular degeneration; CI: Confidence interval; DHA: docosahexaenoic acid; EPA eicosapentaenoic acid; HR: Hazard Ratio
